# Non-Traditional Vectors for Paralytic Shellfish Poisoning

**DOI:** 10.3390/md20080015

**Published:** 2008-06-10

**Authors:** Jonathan R. Deeds, Jan H. Landsberg, Stacey M. Etheridge, Grant C. Pitcher, Sara Watt Longan

**Affiliations:** 1 US Food and Drug Administration Center for Food Safety and Applied Nutrition, 5100 Paint Branch Parkway, College Park, Maryland, 20723, USA; 2 Fish and Wildlife Research Institute, Florida Fish and Wildlife Conservation Commission, 100 Eighth Avenue Southeast, St. Petersburg, Florida, 33712, USA; 3 Marine and Coastal Management, Cape Town, South Africa; 4 State of Alaska Department of Environmental Conservation, Anchorage, AK, USA

**Keywords:** saxitoxins, STXs, paralytic shellfish poisoning, PSP, saxitoxin puffer fish poisoning, SPFP, non traditional vectors, gastropods, crustaceans, puffer fish, public health

## Abstract

Paralytic shellfish poisoning (PSP), due to saxitoxin and related compounds, typically results from the consumption of filter-feeding molluscan shellfish that concentrate toxins from marine dinoflagellates. In addition to these microalgal sources, saxitoxin and related compounds, referred to in this review as STXs, are also produced in freshwater cyanobacteria and have been associated with calcareous red macroalgae. STXs are transferred and bioaccumulate throughout aquatic food webs, and can be vectored to terrestrial biota, including humans. Fisheries closures and human intoxications due to STXs have been documented in several non-traditional (i.e. non-filter-feeding) vectors. These include, but are not limited to, marine gastropods, both carnivorous and grazing, crustacea, and fish that acquire STXs through toxin transfer. Often due to spatial, temporal, or a species disconnection from the primary source of STXs (bloom forming dinoflagellates), monitoring and management of such non-traditional PSP vectors has been challenging. A brief literature review is provided for filter feeding (traditional) and non-filter feeding (non-traditional) vectors of STXs with specific reference to human effects. We include several case studies pertaining to management actions to prevent PSP, as well as food poisoning incidents from STX(s) accumulation in non-traditional PSP vectors.

## 1. Paralytic Shellfish Toxins and Sources

Neurotoxic paralytic shellfish toxins, which comprise saxitoxin and saxitoxin related compounds (STXs), are responsible for the sometimes fatal toxic seafood-related syndromes, paralytic shellfish poisoning (PSP) and saxitoxin puffer fish poisoning (SPFP). These compounds are produced by bloom-forming microalgae – mainly marine dinoflagellates -- approximately ten *Alexandrium* species, *Gymnodinium catenatum*, and *Pyrodinium bahamense* -- and freshwater or brackish cyanobacteria, *Anabaena circinalis*, *A. lemmermannii*, *Aphanizomenon gracile*, *A. issatschenkoi* (as *A. flos-aquae*), *Cylindrospermopsis raciborskii*, *Lyngbya wollei*, *Planktothrix* sp., and *Rivularia* sp. STXs comprise saxitoxin and at least 21 derivatives [1] that in various combinations and concentrations have been associated with PSP. No natural toxigenic dinoflagellate or cyanobacteria population has been found to contain all naturally occurring STX derivatives (Table 1). The toxin profile (i.e., the toxin components produced) is considered by some to be characteristic of the microalgal strain or species [2–3], but this finding has not been consistent among all species in all areas. Some of the STX derivatives are highly toxic (as sodium channel-blocking agents in mammals) and include the carbamate toxins, saxitoxin (STX), neosaxitoxin (NEO), and gonyautoxins (GTX1-4). The decarbamoyl analogues (dcSTX, dcNEO, dcGTX1-4) and the deoxydecarbamoyl analogues (doSTX, doGTX2, doGTX3) are of intermediate toxicity. The least toxic derivatives are the *N*-sulfocarbamoyl toxins, B1 (GTX5), B2 (GTX6), and C1–C4 [1, 4]. Although not usually associated with PSP, *Cochlodinium polykrikoides* (as *Cochlodinium* type ’78) has been shown to produce two unique, zinc-bound, NEO-like compounds [5]. In 1977, *Cochlodinium* sp. was implicated in PSP outbreaks in Venezuela [6], but corroborative evidence is lacking.

Numerous microalgal species have been documented to produce STXs and all are potentially human health risks via the food chain. However, the sources of the majority of PSP reports are the marine dinoflagellates *Alexandrium tamarense*, *A. fundyense*, *A. catenella*, *Gymnodinium catenatum*, and *Pyrodinium bahamense*^1^ [84–85]. Because STXs are also produced by freshwater cyanobacteria, there is a potential for STXs to be transferred through the freshwater food web and pose a risk to human consumers of freshwater products (e.g. mollusks) contaminated by these toxins [86]. STX(s) composition and concentration can vary amongst microalgal species and strains; with geographical location, with environmental factors, and under different experimental conditions [25, 39, 87–88]. Because the toxin profiles of STX-producing dinoflagellate species differ, the exposure dose and the proportion of highly toxic STX derivatives to which animals are exposed will also vary [89–90].

STXs are present in a wide range of aquatic organisms and they have been documented to occur when dinoflagellates were apparently absent [91]. Knowledge of the widespread distribution of STXs and results of a series of experimental studies has led to the conclusion that in some cases dinoflagellates are not the only source of STXs [92]. Although still not definitively proven, a bacterial origin for STXs has been proposed, and bacteria may play a role in the production of STXs in certain dinoflagellate species [22, 92–97].

STXs are highly lethal, having an LD_50_ in mice (intraperitoneally [i.p]) of 10_μg/kg (as compared to an LD_50_ for sodium cyanide at 10 mg/kg [27]. STXs are potent neurotoxins that bind to site 1 on the voltage-dependent sodium channel, block the influx of sodium into excitable cells, and restrict signal transmission between neurons. Symptoms of PSP are paresthesia and numbness, first around the lips and mouth and then involving the face and neck; muscular weakness; sensation of lightness and floating; ataxia; motor incoordination; drowsiness; incoherence; progressively decreasing ventilatory efficiency; and in high doses, respiratory paralysis and death [98–99].

## 2. Traditional Vectors of Saxitoxins to Human Consumers

Most humans who experience PSP have consumed toxic bivalves [84], but occasionally, non traditional vectors such as toxic gastropods and crustaceans [85], and rarely toxic fish [52, 100] are implicated (see section 3). Numerous fatal cases of PSP have been reported globally [101] but the successful implementation of programs monitoring for the presence of both STX-producing microalgae and the presence of STXs in shellfish in many countries has helped to minimize public health risks. To our knowledge, all documented human PSP cases have been caused by toxic marine dinoflagellates; for the most part, the geographical distribution of such PSP outbreaks has been related to the global distribution of the various STX-producing species and their toxigenic strains [90].

Because PSP outbreaks typically result from the consumption of toxic marine shellfish, most studies on STXs concern those vector species that are edible, economic resources. Globally, STXs have been documented in numerous species of mollusks, primarily bivalves, and extensive reviews are available on their toxic occurrence, distribution, exposure, biotransformation, and effects [84, 101–104]. Only a brief literature survey of STXs in traditional bivalve vectors will be provided here.

STX was first isolated from toxic Washington butterclams, *Saxidomus gigantea* [105–106]. In the USA, the first red tide bloom that led to a major PSP outbreak occurred in September 1972 from southern Maine to Cape Ann, Massachusetts. Blue mussels (*Mytilus edulis*) and softshell clams (*Mya arenaria*) were most susceptible to STX(s) accumulation and were the most toxic bivalves. Northern quahogs (*Mercenaria mercenaria*) did not accumulate toxins, even in areas where blue mussels and softshell clams had high STX(s) levels. Eastern oysters (*Crassostrea virginica*) had very low STX levels [107]. In a few isolated areas, softshell clams and blue mussels remained toxic until April 1973 [108], but it is not known whether this was due to slow depuration or a re-occurrence of toxic cells.

The fate and distribution of STXs in bivalves varies according to harmful algal bloom (HAB) characteristics; environmental conditions; prior history of exposure; species, intrapopulation, and individual variability; uptake dynamics and detoxification mechanisms; anatomical localization and retention; physiological breakdown or biotransformation mechanisms; and differences in initial toxicity of dinoflagellates [84, 101, 103–104, 109–115]. Differences between bivalve species in the ability to accumulate STXs have been correlated with each species’ *in vitro* nerve sensitivity to STX and ability to continue actively feeding during toxic blooms [116–117]. Some bivalves demonstrate resistance to STXs [118] contributing to an increased risk of PSP in humans [119].

Bivalves retain STXs for different lengths of time, and the toxic components retained vary; knowledge about these differences aids in the management and prevention of PSP. Some species depurate toxins rapidly whereas others are slow to depurate. A range of STX toxicity levels is found in different bivalve species. Extremely high STX concentrations have been found in the mussels *Mytilus trossulus* and *M. edulis*, in softshell clams and Washington butterclams, and in the scallops *Patinopecten yessoensis* and *Placopecten magellanicus*. In other bivalves, such as northern quahogs and oysters, *Crassostrea* spp., STXs are at low levels or are absent [84, 89, 104]. Depuration times also vary between species. Most species can eliminate STXs within weeks [84, 101], whereas Washington butterclams, sea scallops (*P. magellanicus*), and Atlantic surfclams (*Spisula solidissima*), are known to retain high levels of toxins for long periods of time (from months to more than five years) [102, 109, 120].

The toxin profiles of toxic bivalves and associated PSP risks to human consumers vary depending upon the toxigenicity of the dinoflagellate species to which the mollusks are exposed. For example, in general, bivalves exposed to *Alexandrium tamarense, A. catenella*, and *A. minutum* accumulate high GTX levels, whereas bivalves exposed to *Pyrodinium bahamense* and *G. catenatum* accumulate very low levels of GTX [89]. Bivalve toxin profiles also vary by geographic region, by season, and by the distribution of toxic components in different tissues [2, 102, 109–110, 120–122]. The location and deposition weight of toxin components in the various bivalve organs vary between species. For example, in the scallops *P. magellanicus* and *P. yessoensis*, the majority of the toxins are concentrated in the digestive gland, and while toxicity levels in the gills, gonads, and adductor muscles are typically less than the regulatory action level of 80 μg STXeq/100g, concentrations in gills and gonads have on occasion been above regulatory limits [123]. Since toxins are not readily accumulated in the adductor muscle of scallops, when this is the only part of the shellfish consumed, they are usually considered safe for public consumption, even in the presence of toxic algae [102].

Because they naturally ingest a variety of dinoflagellate species and strains, bivalves are exposed to a variety of toxic components. Knowledge of which toxins are deposited in which tissues and how they are biotransformed at each trophic level may be critical for determining the public health risk associated with the consumption of different shellfish species and their consumable tissues. For example, Atlantic surfclams and sea scallops are naturally exposed in New England to STXs associated with *Alexandrium* spp. STXs are typically stored in the tissues of these species, whereas other potentially poisonous substances such as the carbamate-derivative gonyautoxins are converted to less toxic compounds. The ability to convert carbamate toxins to their corresponding nontoxic decarbamoyl derivatives has been demonstrated in a few bivalves, such as Atlantic surfclams; Pacific littleneck clams, *Protothaca staminea*; and the Japanese clams *Peronidia venulosa* and *Mactra chinensis* [1, 110, 117, 124]. Because of public health concerns and the development of safety protocols, it is critical that we understand the dynamics of toxin distribution in different species, particularly in edible tissues.

## 3. Non-Traditional Vectors of Saxitoxins to Human Consumers

Non-bivalve invertebrates, the primary focus of this review, have increasingly been documented to accumulate STXs [85] and have been implicated in PSP incidents. Amongst the mollusks, apart from traditional bivalve vectors, gastropods (Table 2) and rarely cephalopods (the octopus *Abdopus* sp. [125]), accumulate STXs apparently without any obvious ill effects [126, 127].

### 3.1 Gastropods

Molluscan gastropods including oysterdrills, volutes, whelks, periwinkles, moon snails, conch, slipper limpets, and turban shells (Table 2) accumulate STXs primarily acquired through predation (in many cases of toxic bivalves) [85, 128].

Because gastropods are able to bioaccumulate high concentrations of STXs, they are a significant risk to human consumers, and have been the cause of multiple fatalities, particularly in the Far East (Table 2). In gastropods, STXs are typically concentrated in the digestive gland but some species such as the moon snail, *Lunatia heros*, concentrate toxin in the muscle tissue [85]. Variability in toxicity is also a function of species differences in predatory habits, differential acquisition of toxins by individuals, sporadic feeding, their ability to move away from toxin sources, and because gastropods are slow to depurate toxins [85].

#### 3.1.1 Case Study 1: STXs in Abalone

Unlike filter-feeding bivalve mollusks, gastropods such as abalone (other common names: ormer and perlemon) feed by scavenging, predation, and grazing. Their diet primarily consists of kelp and other seaweeds, making them unlikely candidates for PSP. However, there have been reports of PSP toxins in abalone off the northwest coast of Spain [83, 148–149] and the west and south coasts of South Africa [150].

##### Spain

STXs were first detected in the Galician abalone *Haliotis tuberculata* in 1991. Subsequently, abalone in this region was affected by toxin concentrations sufficiently high enough to enforce indefinite closure of the industry in 1993 [148]. dcSTX was the most abundant toxin reported in abalone, followed by low concentrations of STX [83, 148–149]. The source of these toxins remains unknown. The dinoflagellates *Gymnodinium catenatum* and *Alexandrium minutum* are the common STX(s) producers in this region; however, they do not display temporal or geographical distributions corresponding to that of abalone toxicity [83, 151]. Also, the toxin profile of these potential sources differs from that of the abalone; although biotransformation may be responsible for this discrepancy. The authors postulated that cyanobacteria may be the source of the toxin and they report measurable STX(s) concentrations for the cyanobacterium *Rivularia* sp. It is noteworthy that no other PSP problems were reported for other mollusks or crustaceans in this region [149]. Anatomical distribution showed high toxicity in the epipodial fringe [149, 151] with as much as 2.6 times more toxin in the epithelium compared to the foot. Toxicity generally increased with increasing abalone size. Depuration of toxin in abalone did not occur during three months of monitoring cultured abalone fed a variety of macroalgae [83]. No other abalone PSP reports have been reported for this area in the recent literature. However, there was a report by Huchette and Clavier (2004) [152] that indicated the abalone fishery reopened in Spain in 2002, but was closed again shortly thereafter due to an oil spill.

##### South Africa

Abalone harvesting represents an old fishery in South Africa and currently this fishery includes recreational, subsistence and commercial harvesting. In addition to wild harvest, the 1990’s represented a period of movement towards land-based abalone farms. In 1999, STXs were detected in abalone from two farms located along the west coast of South Africa [150]. Subsequent testing also found the presence of toxins in wild abalone. Throughout this evaluation of abalone PSP, toxicity was tested using the AOAC mouse bioassay with levels reported from below the limit of detection to greater than 1600 μg STX eq/100 g. For most of these cases, analysis was conducted on the entire animal; however, some samples were separated into specific body parts to examine anatomical distribution. As with other organisms, there appears to be large variability in toxicity between individuals (5–10 fold variability reported). Observations of detached and paralyzed abalone in the wild were made and analysis confirmed the presence of STXs. Pitcher *et al.* [150] found a geographical gradient in toxicity with the highest toxicity observed in abalone from the north and a general decrease southwards. The notable distinction in toxin composition for South African abalone compared to those from Spain is that only STX was detected in the former [150]. This profile is different from the known STX(s) source (*Alexandrium catenella*) and other vectors (e.g. mussels *Mytilus galloprovincialis*) in the area [150, 153]. Given the toxin profile differences and the feeding behavior of abalone, it is uncertain what the source of STXs is to the abalone. Further investigations by Etheridge *et al.* [154] indicated the putative source of toxins to be from their natural diet, the macroalgal kelp *Eklonia mamixa*. Depuration studies suggest that either abalone can retain the toxins for long periods of time or the toxin was still present during the studies. Pitcher *et al.* (2001) [150] found that abalone retained toxins for at least seven months with no apparent decline in toxicity when kept under controlled laboratory experiments with kelp as the diet. Controlled feeding experiments [154] were conducted using juvenile abalone (2 cm in length, average wet weight 0.6 ± 0.3 g) and demonstrated that depuration did not occur when abalone were either fed kelp or were starved. However, depuration rates of 6.3 μg per 100g per day were observed when abalone was fed artificial feed. Initial toxicity in the abalone was 160 ± 38 μg STX eq per 100g and after being fed artificial feed for two weeks toxin levels decreased to 72.3 ± 12.5 μg STX eq per 100. Thus, it is possible that feeding farmed abalone artificial feed prior to market could reduce the risk of PSP. Toxin distribution among abalone tissues demonstrates differential uptake and compartmentalization. Thus, the contribution of each tissue to total toxin burden is a function of both its absolute toxicity and relative weight contribution. Pitcher *et al.* [150] found moderate amounts in the foot and viscera and high amounts in the epipodial fringe. Given the high surface area of the epipodial fringe, it contributes significantly to the total toxin burden. Abalone is often marketed with the foot for human consumption; therefore, it has been suggested that scrubbing and/or removing epithelial tissue could decrease toxicity to safe levels for consumption. This could potentially be used as a strategy to reduce toxin levels prior to market.

Periodic PSP events still occur along the west coast of South Africa. In many cases this has resulted in prevention of exporting live abalone. However, shucking and scrubbing (i.e. removing the epithelial layer of the abalone) decreased toxicity to safe levels (aggregate toxicity < 80 μg/100 g whole animal). For example, Pitcher *et al.* [150] found that toxicity levels in the foot and epipodial fringe (one of the largest reservoirs of STXs containing > 800 μg/100 g in some cases) both decreased significantly (approximately 6 to 9-fold) when scrubbed. Currently, testing for toxins is done regularly under the South African Shellfish Sanitation Program run by Marine and Coastal Management under an MOU. When traces of toxin are detected, sampling frequency increases and farms in the affected area can be prevented from exporting. Again, shucking, scrubbing and cleaning remain processing options (e.g. canning) that can be used to safely market abalone from this region.

#### 3.1.2 Case Study 2: STXs in Whelks and Moon Snails

##### Japan

During surveillance on the toxicity of invertebrates such as bivalves inhabiting the coasts of Hiroshima Bay in 2001 and 2002, the carnivorous gastropod rapa whelk *Rapana venosa,* collected in the estuary of Nikoh River, was found to contain toxins which showed paralytic actions in mice; the maximum toxicities (as STXs) were 4.2 MU/g (May 2001) and 11.4 MU/g (April 2002). This equated to total toxicities of 224 and 206 MU/viscera for these specimens (1MU = 0.18 μgSTX). In an attempt to identify the toxic principle(s) in this gastropod, the viscera were extracted with 80% ethanol acidified with acetic acid, followed by defatting with dichloromethane. The aqueous layer obtained was treated with activated charcoal and then applied to a Sep-Pak C18 cartridge. The unbound toxic fraction was analyzed by high-performance liquid chromatography. The gastropod toxin was rather unexpectedly identified as STXs. It was comprised of GTX3, GTX2, and STX as the major components, which accounted for approximately 91 mol% of all components along with STXs Cl and C2, which are N-sulfocarbamoyl derivatives. Judging from their toxin patterns, it was suggested that the STX(s) toxification mechanism of the gastropod was phytoplankton, such as *Alexandrium tamarense*, transferred to and accumulated in filter-feeders such as the short-necked clam, and then transferred to this carnivorous whelk through predation [137].

##### New England, USA

Several species of moon snail and whelk are also known to accumulate STXs and such gastropods are often prohibited for harvesting in waters of the states of Maine and Massachusetts, USA. Closures in waters off the coast of Maine are made by the Department of Marine Resources and are posted on their website (http://www.maine.gov/dmr/rm/public_health/closures/-pspclosures.htm [accessed 3 March, 2008]). The moon snail of interest in this area is *Lunatia heros*, and the whelks impacted by closures are of the family Muricidae and Buccinidae. In Maine state waters, harvesting of moon snails and the whelk *Buccinum undatum* is closed as a precaution whenever the blue mussel *Mytilus edulis* exceeds the regulatory limit for STXs, due to the observation that if there are any bivalves carrying STXs then any co-occurring carnivorous gastropods will be toxic as well (D. Couture, pers. comm.). The Division of Marine Fisheries is responsible for the safety of seafood harvested in Massachusetts state waters and their closures can be found on their website (http://www.mass.gov/dfwele/dmf [accessed 3 March, 2008]). Off the coast of Massachusetts, closures are often in effect for the lobed moon snail *Polinices duplicatus* and the northern moon snail *L. heros*, as well as the channeled whelk *Busycon canaliculatum* and the knobbed whelk *B. carica* (M. Hickey, pers. comm.). Notably, harvesting closures are often extended for moon snails longer than for other species because they accumulate higher levels of toxin by feeding on toxic bivalves. Certain carnivorous mollusks also appear to retain toxins for longer periods of time than the source bivalves. For example, an extensive *Alexandrium fundyense* bloom occurred off the coast of New England in 2005 resulting in PSP closures of vast regions in state and federal waters [155]. The U.S. Food and Drug Administration is responsible for the safety of seafood harvested in federal waters and they began sampling shellfish in the impacted areas during the 2005 bloom. Sampling continued in 2006 and toxicity levels above the action level were still being detected for moon snails and whelk from federal waters off the coast of Massachusetts (Table 3). In that region, the only other species that remained toxic was the sea scallop (*P. magellanicus)*, in the viscera (Table 3). Sea scallops are known to retain toxins in viscera for long periods of time compared to other co-occurring species [104]. These data demonstrate the need to monitor toxicity for these non-traditional seafood products, even after bloom conditions have dissipated.

### 3.2 Crustaceans

Among non-filter feeding, non-molluscan species, STXs have been found most commonly in xanthid crabs (Table 4) [156–159]. In some cases, toxins were believed to be derived from the calcareous alga *Jania* sp., consumed by the crabs [160]. STXs have also been found in other crab species, lobsters, crayfish, penaeid shrimp, barnacles (Table 4) and a few other crustacea [85, 147].

Many macrocrustaceans, including lobsters, are able to tolerate and hence concentrate extremely high levels of STXs. Lobsters can accumulate STXs by preying on, among other species, blue mussels which can have maximum toxicities of up to 23,000 μg STX eq/100g [162]. Jiang *et al.* (2006) [175] demonstrated the transfer and metabolism of STXs from the scallop *Chlamys nobilis* to spiny lobsters *Panulirus stimpsoni*. When experimentally fed with toxic viscera of *C. nobilis*, the hepatopancreas of *P. stimpsoni* showed the same toxin profile as that of the scallop, including GTX1–3, C1+C2 and B1, and dcGTX2+3. In spiny lobsters depurated with non-toxic squid, the mildly toxic N-sulfocarbamoyl toxins tended to transform into more highly toxic carbamates. After being fed toxic *C. nobilis* for six days, spiny lobsters selectively accumulated N-sulfocarbamoyl toxins with low toxicity. The concentration of dcGTX (2+3) in *P. stimpsoni* decreased significantly and was not detectable after six days depuration, which was likely due to their initial low level of toxicity.

Xanthid crabs can harbor toxins [176] in their tissues at concentrations (Table 4) that would be fatal to other animals [177]. Maximum toxin levels of more than 16,000 μg STX eq/100g were found in the xanthid crab *Atergatis floridus* in Australia, even though the majority of samples contained less than 80 μg STX/100g [161]. In Japan, an individual *Zosimus aeneus* contained nearly 16,500 Mouse Units (MU) per g [178], which is equivalent to 300,000 μg STX eq/100g [105, 161]. Several species of xanthid crabs produce a hemolymph protein, saxiphilin, that binds with STX and which may confer some resistance to possible toxic effects [177]. This mechanism may explain why some xanthid crab species appear to tolerate exceptionally high levels of toxins [177]. When a mixture of GTX2 and GTX 3 in 3% NaCl was injected into the right chela of *A. floridus*, the rate of dissipation within the crab was fairly high and suggested that high concentrations of toxin are not accumulated in all species [179].

#### 3.2.1 Case Study 3: STXs in Crabs

##### East Timor

In October 2000, an adult male died within hours of ingesting a xanthid crab *Zosimus aeneus* (Xanthidae) [172]. A second, yet uneaten specimen of *Z. aeneus* from the same meal contained a total toxicity of 162.8 μg STX eq/100g tissue (comprising GTX2, GTX3, NEO, dcSTX, and STX); these same toxins were identified in the gut contents, blood, liver and urine of the victim. Metabolism of STXs occurred with the ingested crab harboring GTX2, GTX3 and STX, whereas NEO, dcSTX and STX dominated the STXs in the victim's urine. The STX(s) composition in the gut contents, in both their identity and proportion, was intermediate between the eaten crab and the urine suggesting that toxin conversion commenced in the victim's gut. The victim's meal did not consist solely of the toxic crab eaten and the possibility of other food items acting in a synergistic manner with the consumed STXs cannot be discounted. As well as STXs, xanthid crabs are known to harbor tetrodotoxin (TTX) and palytoxin [180–182].

##### Japan

Oikawa *et al.* [163–164, 183–184] showed that the edible crab *Telmessus acutidens* both accumulated and retained STXs after consuming contaminated mussels (*Mytilus galloprovincialis*) in Japan. STXs in two shore crab species, *T. acutidens* and *Charybdis japonica*, were compared with the toxin in the prey mussel *M. galloprovincialis* and causative dinoflagellates *Alexandrium tamarense*, all having been collected at Onahama, Fukushima Prefecture, in the northern part of Japan. When the toxicities were detected in mussels by mouse bioassays, 73.7% of the sampled *T. acutidens* were toxic in the hepatopancreas. *Charybdis japonica* was also expected to be a possible vector species, but only small quantities of STXs were detected in eight specimens of the crab by HPLC analysis. The difference in STX(s) accumulation in both *T. acutidens* and *C. japonica* was then investigated at Onahama, Fukushima Prefecture, from 2002 to 2005. The level of toxin accumulation in the hepatopancreas of *T. acutidens* corresponded to that of mussels when examined on a yearly basis. In 2003, some crabs had a high toxicity of approximately 1000 MU, which compares to one-third of the human minimum lethal dose. Therefore, it was concluded by the authors, that *T. acutidens* should be monitored as a vector species of PSP toxins. The toxin profile of *T. acutidens* was also investigated. Because an increase in highly toxic species of STXs with a decrease in low toxic species, such as *N*-sulfocarbamoyl-11-hydroxysulfate toxins, was not clearly observed between consecutive samples, toxin transformation in *T. acutidens* was considered to have a minimal impact on toxicity. STXs were also detected in several specimens of *C. japonica*, but the highest toxicity was only 7.4 MU/g in the hepatopancreas. Lastly, accumulation and depuration rates of STXs in the crab *T. acutidens* were investigated by feeding toxic and non-toxic mussels under laboratory controlled conditions. The crab accumulated toxins in the hepatopancreas in proportion to the amount of toxic mussels they ingested, and the toxicity in the crab hepatopancreas became 3.2 fold of that in the prey mussels after 20 days of feeding. During depuration, a fast reduction of the total toxicity was observed in the crab, and the retention rate of the toxicity after five days depuration with feeding of non-toxic mussels was 45.8 +/− 18.7%. The reduction of the toxicity was moderated in the later period of depuration, and the retention rates of the total toxicity after 10 and 20 days were 54.1 +/− 29.8% and 14.5 +/− 9.0%, respectively. The toxin profiles in the crab and mussel were investigated by high performance liquid chromatography, and reductive conversions of the toxins were observed when the toxins were transferred from the mussel to the crab. Consequently, high concentrations of GTX2, GTX3, and STX that were not detected in the prey mussels were found in the crab.

##### Alaska, USA

**Figure 1:**
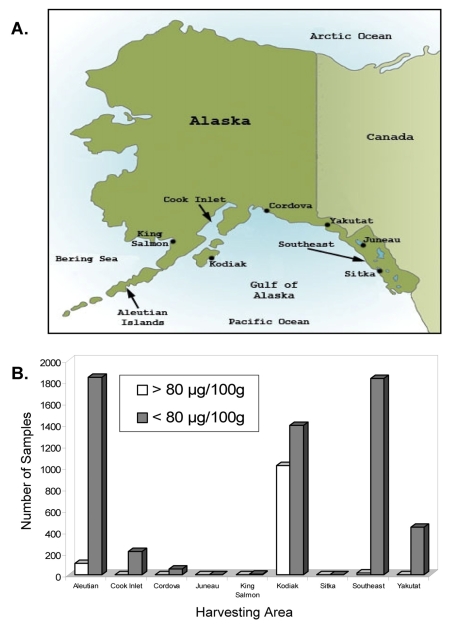
**(A)** Map of the state of Alaska, U.S.A. indicating collection sites for crab STX testing. **(B)** Number of samples above and below the regulatory action limit of 80 μg STX eq./100 g tissue for all species of commercially harvested crab in Alaska between 1992–2004, broken down by major testing area. All values are for crab viscera only. Sample = 1 crab. All testing done by AOAC mouse bioassay.

**Figure 2:**
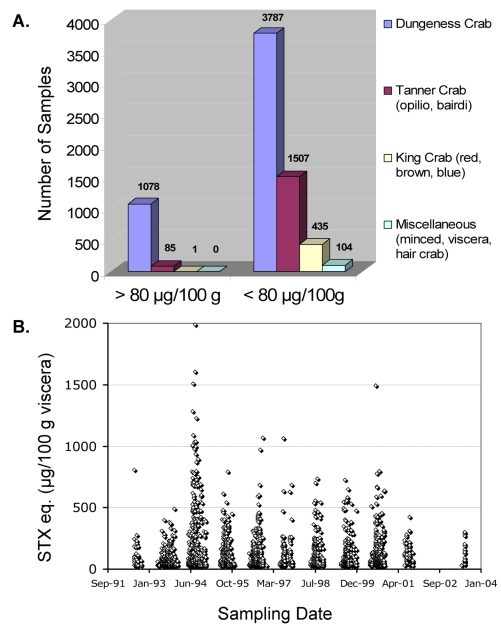
**(A)** Number of samples above and below the regulatory action limit of 80 μg STX eq./100 g tissue for commercially harvested crab in Alaska between 1992–2004 for all testing areas, broken down by crab type: Dungeness (*Cancer magister*), Tanner: (*Chionoecetes opilio* and *Chionoecetes bairdi*), King Crab: (Red, *Paralithodes camtschaticus*; Blue, *Paralithodes platypus*; Brown, *Lithodes aequispinus*), and Miscellaneous (including minced, viscera, and Hair Crab, *Erimacrus isenbeckii*). **(B)** Total STXs (in μg STX equivalents/100 g viscera) for all commercially harvested crab species in Alaska for all areas from 1992–2004.

Although not thoroughly recorded in the scientific literature, the State of Alaska, Division of Environmental Health, Food Safety and Sanitation Program has been observing elevated levels of STXs in viscera from several species of commercially harvested crabs for years (Figure 1). PSP is endemic to the coastal communities of the State of Alaska [185]. The high frequency of STX producing dinoflagellates coupled with an extensive seafood harvesting industry prompted the state to establish a STX monitoring program. Most commercially harvested crab in Alaska is landed in the open waters of the Bering Sea, but limited harvesting does occur in areas where PSP toxicity is commonly seen in filter-feeding bivalves. In these areas, high regional and species variability in crab STX(s) content exists, with Dungeness crab (*Cancer magister*) from Kodiak Island appearing to be a consistent food safety concern (Figure 1, 2). To protect public safety, the State of Alaska Food Safety and Sanitation Program, Department of Environmental Conservation, and the Department of Fish and Game perform both pre-season environmental sampling and in season monitoring of both harvesting areas and harvested product. A conservative action level of 70 μg STX eq. /100g viscera (FDA regulatory action level = 80 μg STX eq. /100g tissue) has been established above which product cannot be marketed either live or whole cooked but must be eviscerated at the processing facility where it is landed (http://www.dec.state.ak.us/eh/fss/seafood/PSP/dungeness.htm [accessed 3 March, 2008]). Due to the success of this monitoring program, no reports of PSP due to the consumption of commercially harvested crab have been reported even though visceral concentrations exceeding 500 μg STX eq./100 g have been observed almost yearly in some areas (Figure 2b).

### 3.3 Other invertebrates

Other, non-molluscan invertebrates that accumulate STXs include annelid tubeworms *Eudistylia* sp. [161], and echinoderm starfish *Asterias amurensis*, *Astropecten scoparius*, *A. polyacanthus*, and *Pisaster ochraceus* [161, 186–187]. Thus far, these species have not been implicated in PSP cases.

### 3.4 Fish

Although not usually targeted, STXs have been incidentally found in numerous species of fish (Table 5). As with shellfish, because STXs are water soluble compounds, researchers believed that cold-blooded vertebrates such as finfish did not typically accumulate STX(s) nor were fish negatively affected by STXs [131]. However, the transport of STXs through the food chain and the vectoring and accumulation of toxins through zooplankton have been identified as important mechanisms by which toxins become available to higher trophic levels such as fish [188–193].

As they are in bivalves, the toxic profiles of STXs that accumulate in fish are likely to be partially determined by species-specific differences in the bioconversion process or are dependent upon the variety and toxin profiles of their toxic prey species. During July 1988, a small bloom of *Alexandrium fundyense* occurred in southwestern Bay of Fundy, New Brunswick, Canada. The highest concentration in a surface-water sample was 7.5 x 10^3^ cells/L. Concentrations of STXs in Atlantic mackerel, *Scomber scombrus*, liver extracts were measured by mouse bioassay and ranged from 40–209 μg STXeq/100g wet weight. By far the dominant component in mackerel liver was STX except in a few fish where NEO was also dominant. GTX2 and GTX3, and rarely B2, were also detectable. The difference between the toxic profiles of the fish and *A. fundyense* was attributed to the variety of toxic prey consumed by the fish. The fact that mackerel accumulate STXs demonstrates the transfer of these toxins up the food chain [194–195, 207]. Atlantic mackerel in the Gulf of Lawrence retained STX, GTX2, and GTX3 all year round and progressively accumulated STXs throughout their life, likely vectored via zooplankton feeding on toxic *Alexandrium* [195].

Fish, with the exception of puffer fish (see case study 5 below) are not usually vectors for STX(s) transfer if humans only eat the muscle. Accumulation of STXs is usually confined to the fish’s gut, and either certain species perish before detectable amounts of toxin appear in the muscle [207–208] or negligible concentrations of toxins accumulate in the muscle. In experimental studies, several fish species challenged with oral (LD_50_ = 400–750 μg/kg body weight) or i.p. (intraperitoneal) (4–12 μg/kg body weight) doses of STX showed similar symptoms: loss of equilibrium; gasping; reduced locomotor activity; short, irregular, hyperactive periods; and death within one hour. Heavy accumulation of STX was confined to the gut (340–840 μg per 100g tissue), while STX occurred in the muscle tissues at a level an order of magnitude lower than in the gut [208]. Kwong *et al.* [209] exposed green-lipped mussels *Perna viridis* and black sea bream *Acanthopagrus schlegeli* to toxic *Alexandrium fundyense* to evaluate the accumulation, distribution, transformation, and elimination of STXs in controlled experimental conditions. Mussels were fed *A. fundyense* for seven days followed by three weeks of depuration, and the fish were fed toxic clams for five days followed by two weeks of depuration. The fish viscera accumulated most of the STXs. In the fish, the ratio of C1/C2 was 3.0 times (*p* < 0.01) higher when compared to the mussel tissues, indicating that conversion from C2 to C1 might have occurred when the toxin was transferred from the clams to the fish. Jiang *et al.* [210] investigated the transmission and transformation of STXs from *A. tamarense* to the cladoceran *Moina mongolica* and subsequently to the larval fish *Sciaenops ocellatus*. STXs were transferred to *S. ocellatus* when they preyed upon STX(s)-containing *M. mongolica*. During the experimental period, *A. tamarense*, *M. mongolica* and the larval fish’s digestive glands contained C1 and C2 toxins, while the viscera of *S. ocellatus* contained NEO. The proportion of C2 to C1 toxins increased when STXs were transferred from *A. tamarense* to *M. mongolica*, but in the subsequent transfer from *M. mongolica* to *S. ocellatus* the proportion of C1 to C2 toxins increased. During depuration, the contents of C1 and C2 toxins in fish larvae decreased with the duration of depuration, but NEO remained relatively constant. The present results indicated that, using a cladoceran as the vector, STXs can be transferred from toxic algae to a high trophic level fish and metabolized in the fish. Future work should address the metabolic characteristics of STXs in cladocerans and the end result when they are transferred to fishes.

#### 3.4.1 Case study 4: STXs in planktivorous fish

##### Far East

With the puffer fish exception, because STXs do not typically accumulate in fish muscle, humans who consume only the muscle are unlikely to become intoxicated. However, those those who consume whole fish and eat the viscera are likely to become sick. In 1976 in Brunei, 14 nonfatal PSP cases were associated with the consumption of the planktivorous fish *Rastrelliger* sp. during a bloom of *Pyrodinium bahamense* var. *compressum* [48]. One PSP incident in 1983 in Indonesia involved 191 cases and four human fatalities due to the consumption of the planktivorous clupeoid fish *Sardinella* spp. and *Selaroides leptolepis*. In a second incident in November 1983, 45 people became ill after consuming fish and suffered numbness, dizziness, and tingling sensations of the lips, tongue, and throat. Although no known toxic dinoflagellate was associated with the event [100], PSP was highly suspected [211]. STXs with toxin profiles similar to *Pyrodinium bahamense* have been confirmed in gut contents of *Sardinella* sp. from Brunei [41] and in PSP incidents involving *Pyrodinium*, toxic shellfish, and fish that were reported from the Philippines [211]. These incidents likely occurred because it is customary in south-east Asia to eat small fish whole, including any potentially toxic viscera [211–212].

#### 3.4.2 Case study 5: STXs in puffer fish

One exception to the general rule that STXs tend not to accumulate to levels associated with human intoxication in fish muscle is in members of the family Tetraodontidae (puffer fish) (Table 5) inhabiting marine and freshwater habitats. STX was first described as a minor component of highly toxic (with TTX) *Takifugu pardalis* livers in Japan [201]. Soon after, STX was confirmed as a minor component in the additional Japanese species *T. poecilonotus* and *T. vermicularis* [202], and as a major toxin in *Arothron firmamentum* [197]. STXs were found to be the sole toxic component in a range of freshwater puffer fish, some responsible for human poisoning events, in Thailand, Bangladesh, Brazil, and in Cambodia (Table 5). Seven species of marine puffer fish in the Philippines (Table 5) were found to contain both STXs and TTX, with STXs being the dominant toxin in several species [198].

##### Florida, USA

Puffer fish became an important source of protein on the east coast of the United States during the Second World War, and supported a commercial fishery in the decades that followed. The primary species landed was the northern puffer fish (*Sphoeroides maculatus*) but limited numbers of the southern puffer (*S. nephelus*), primarily from Florida, was also harvested [213]. The industry was centered in the mid-Atlantic states of Virginia, Maryland, New York, and New Jersey with > 6,000 metric tons landed in 1965 (National Marine Fisheries Service Statistics and Economics Division, personal communication). Fish were marketed dressed and skinned under the name “sea squab”. Although the commercial puffer fish industry has steadily declined since the 1970’s, today being only harvested as by-catch, domestic puffer fish can still be found in some U.S. fresh fish markets. In addition, an average of > 500,000 fish was caught annually between 1981 and 2003 by recreational anglers in the U.S. where they are easily obtained by a range of gear including hook and line (National Marine Fisheries Service Statistics and Economics Division, personal communication [213]).

In January 2002, the poison control center in Tampa, Florida, USA received a report of a man hospitalized with symptoms of numbness and tingling of the hands, vomiting, and diarrhea after consuming puffer fish caught during a recreational fishing trip near Titusville, located on the northern Indian River Lagoon (IRL) on Florida’s central east coast [214]. After additional reports of patients with symptoms of neurological illness from Virginia and New Jersey, all associated with what was believed to be *S. nephelus* originating from the northern IRL, uneaten fish muscle samples from the New Jersey incident sent to the Canadian Institute for Marine Biosciences by the New Jersey Department of Health were, surprisingly, found to contain no detectable TTX but to contain significant amounts of STX, with lesser amounts of the STX congeners B1, and dcSTX [215]. This same combination of toxins was confirmed in meal remnants from two separate poisoning events in 2004 [216]. In total, 28 cases of SPFP were reported from 2002–2004 -- all due to fish originating from the northern IRL [58]. These were the first reports of STXs both in Florida marine waters and in indigenous puffer fish in the U.S. In April 2002, the Florida Fish and Wildlife Conservation Commission (FWC) placed a ban on the commercial and recreational harvesting of all puffer fish species for the entire IRL. At the same time, the FWC initiated intensive sampling for STXs in multiple species of aquatic biota in Florida’s coastal waters with emphasis on the IRL. Partial results of this sampling were reported [58, 217]. Analysis of IRL puffer fish found concentrations of STXs in muscle often well in excess of the 80 μg STX eq./100 g tissue regulatory action limit set for shellfish. After extended monitoring, STX concentrations in puffer muscle in certain regions of the IRL remained well above the action limit. As a result, the puffer fishing ban in the IRL was made indefinite in June 2004. Based on toxin profiles and abundance in the IRL during the first SPFP reports in 2002, Landsberg *et al.* [58] suggested the dinoflagellate *Pyrodinium bahamense*, not reported to produce STXs in Florida waters prior to 2002, as the putative toxin source.

Deeds *et al.* [218] confirmed that *S. nephelus* from the northern IRL contained elevated concentrations of STX in muscle (1770 ± 159 μg STX/100g tissue) compared to liver (609 ± 432 μg STX/100g tissue), with only low to non-detectable amounts of TTX in all tissues tested. The additional IRL puffer species *S. testudineus* (checkered puffer) and *S. spengleri* (bandtail puffer), known to only occur further south in the lagoon system, were found to contain significantly greater concentrations of TTX compared to STX in all tissues (maximum concentration for TTX found in *S. testudineus* livers 6076 ± 3283 μg TTX/100g tissue – maximum concentration of STX found in *S. spengleri* livers 74 ± 42 μg STX/100g tissue). This work confirmed *S. nephelus*, a species not associated with toxicity in the IRL prior to these events*,* as the likely cause of all 28 cases of SPFP originating from the IRL during 2002–2004. These events on the east coast of the U.S. represented the first confirmed cases of puffer fish poisoning due solely to STX in North America.

## 4. Conclusion

In comparison to non-traditional (i.e. non-filter feeding) vectors for PSP, more is known about STX sources, routes of exposure, species specific and population specific sensitivities, depuration rates, compartmentalization, and biotransformations in filter-feeding bivalves. As a result, monitoring and management of traditional bivalve vectors for PSP are, in many cases, highly successful and result in the protection of public health. Due to a lack of basic knowledge on the source(s) and fate of STXs in non-traditional vectors, human intoxications due to the consumption of these species are often more unpredictable, and resource closures are often longer and sometimes indefinite. With the apparent expansion in STX producing microrganisms world-wide, an ever-increasing demand for seafood, and the emergence of seafood as an economic commodity for export, particularly in developing countries, more study is required on STX sources, distribution, and fate in these non-traditional PSP vectors to assure both public safety and consumer confidence on local, national, and international scales.

## Figures and Tables

**Table 1:** Microalgal sources of saxitoxins and saxitoxin derivatives (no reference is made to other toxins produced by these species).

Species	Saxitoxin and derivatives	References
**Dinoflagellates**		
*Alexandrium acatenella*	STX	7–9
*Alexandrium andersoni*	STX, NEO	10–11
*Alexandrium angustitabulatum*	unknown toxin composition	12
*Alexandrium catenella*	STX, GTX1–4, NEO,B1–2, C1–4	13–21
*Alexandrium cohorticula*	STX, GTX1–4	22–23
*Alexandrium fundyense*	STX, NEO, GTX1–4, C1–2, B1	9, 24–25
*Alexandrium minutum* (*= A. lusitanicum*)	GTX1–4	20, 26–30
*Alexandrium ostenfeldii*	GTX2–3, B2, C1–2	31–34
*Alexandrium tamarense*	STX, NEO, GTX1–4, B1, C1, C2, C4	9, 21, 35–40
*Alexandrium tamiyavanichi*	STX, GTX1–4, B1, C1–4	41–42
*Cochlodinium polykrikoides* (*= C. heterolobatum, Cochlodinium type’78*)	zinc-bound carbamoyl hydroxy NEO	5
*Gymnodinium catenatum*	STX, NEO, trace GTX2–3, B1–2, C1–4	30, 41, 43–46
*Pyrodinium bahamense*	STX, NEO, B1–B2	41, 47–58

**Cyanobacteria**		
*Anabaena circinalis*	STX, GTX1–4, C1–C2, dcGTX2–3	3, 59–66
*Anabaena lemmermannii*	STX	67
*Aphanizomenon gracile*	STX, NEO	68
*Aphanizomenon issatschenkoi* (as *A. flosaquae*)	NEO, STX	69–77
*Cylindrospermopsis raciborskii*	STX, NEO, GTX2–3	78–79
*Lyngbya wollei*	dcSTX, dcGTX2–3, acetylated STX analogues	80–81
*Planktothrix* sp.	STX	82
*Rivularia* sp.	GTX2, GTX4	83

**Table 2:** Maximum STX concentrations, microalgal sources, and global PSP reports in gastropods.

Gastropod species and presumptive microalgal source	Common name	Maximum STX(s) concentration	Incident	Location	Reference
***Alexandrium acatenella***					
*Polinices lewisii*	Lewis moon snail	176–600 μg STX eq./100g tissue		British Columbia, Canada	129

***Alexandrium catenella***					
*Adelomelon ancilla*	Volute	toxic		Chile	85
*Argobuccinum* sp.	Whelk	Stomach 5629 μg STX eq./100g tissue; Muscle 92 μg STX eq./100g tissue			
*Concholepas concholepas*	Barnacle rock shell	toxic			
*Trophon* sp.	Trophon	toxic			
*Nassarius* sp.	Nassa mud snail (dog whelk)	9 μg STX eq./100g tissue		Washington, USA	85
*Neptunea* spp.		200–250 MU* 100 g^−1^ whole individuals		Alaska, USA	85
*Thais* sp.	Oyster drill	23 μg STX eq./100g tissue (GTX 2 and GTX 3 only)		Washington, USA	85
*Thais lamellosa*	Oyster drill	Whole animal positive			
*Thais lima*	Oyster drill	Whole animal 180 μg STX eq./100g tissue			

***Alexandrium tamarense***					
*Littorina sitkana*	Sitka periwinkle	Trace whole animal		Washington, USA	85
*Lunatia heros* (as *Polinicies heros)*	Northern moon snail	1450 μg STX eq./100g tissue	2 cases PSP	Massachusetts, USA	130
*Buccinum undatum*	Waved whelk	whole body 608 μg STX eq./100g tissue; digestive gland 1600 μg STX eq./100g tissue 3337 μg STX eq./100g tissue	12 cases PSP, 4 fatalities Illnesses and deaths	Quebec, Canada Gulf of Maine, USA	85, 131 85, 132
*Crepidula fornicata*	Slipper limpet	46–58 μg STX eq./100g tissue			
*Colus stimpsoni*	Stimpson’s colus	toxic			
*Lunatia heros* (*=Euspira heros, Polinices heros*)	Northern moon snail	2922 μg STX eq./100g tissue			
*Neptunea decemcostata*	Ten-ridged whelk	Raw~3000–4000, steamed 1060 μg STX eq./100g tissue			
*Thais lapillus*	Purpura	34 μg STX eq./100g tissue			
*Lunatia heros* (*=Euspira heros, Polinices heros)*	Northern moon snail	247 μg STX eq./100g tissue		Gulf of St. Lawrence, Canada	133
*Adelomedon brasiliana*	Volute	28 MU g^−1^ whole		Argentina	134
Zidona angulata*	Volute	210 MU g^−1^ viscera; 25 MU g^−1^ foot; 17 MU g^−1^ mucus	Mild case of PSP		
*Busycon* spp.	Whelk	50–500 MU 100 g^−^^1^		Quebec, Canada	85

*Rapana venosa*	Veined rapa whelk	11.4 MU g^−1^ viscera		Hiroshima Bay, Japan	135

***Gymnodinium catenatum***					
*Haliotis tuberculata*	Abalone	467 μg STX eq./100g muscle		Spain	83

***Pyrodinium bahamense***					
*Lambis lambis*	Spider conch	ND – 175 MU 100 g^−1^ whole	Several PSP cases	Sabah, Malaysia	136–137
*Oliva vidua fulminans*	Olive	2525 MU 100 g^−1^ whole	5 human fatalities; 8 cases of PSP	Malaysia	136–138
*Natica* sp.**	“Tekuyong”	71–876 MU 100 g^−1^		Borneo	139–140
**Unknown origin**					
*Nassarius siguijorensis*	Nassa	370 MU 100 g^−1^		Daya Bay, Guangdong Province	141
*Nassarius succinctus*	Nassa		68 cases of PSP, March–Aug 1979; 1 fatality and 7 hospitalized	Zhejiang Povince, China	128, 142
*Nassarius* spp.	Nassa		50 PSP cases, 3 fatalities, April– May 2002 55 PSP cases, 1 fatality; summer 2004	Fujian Province, China Yin Chuan city, China	128 128
*Nassarius* spp.	Nassa	107,413 MU 100 g^−1^		Zhoushan Islands, China	128
*Charonia lampas*	Trumpet shell	17.5 MU g^−1^ digestive gland		Galicia, Spain	143
*Natica lineata*	Lined moon shell	PSP toxins		Taiwan	144
*Natica vitellus*	Calf moon shell				
*Niotha clathrata*	Basket shell	PSP, GTX-3			144–145
*Neptunea arthritica*	Arthritic neptune	GTX 1–4, neoSTX, STX		Sanriku coast, Japan	146
*Tectus fenestratus*	Fenestrate top shell	18.7 μg STX eq./100g tissue		Northwest Australia	147
*Tectus nilotica maxima*	Top shell	5.0 MU g^−1^ whole		Ishigaki Island, Japan	52
*Tectus pyramis*	Top shell	19 MU g^−1^ whole		Ishigaki Island, Japan	52
*Turbo argyrostoma*	Turban shell	20 MU g^−1^ whole		Ishigaki Island, Japan	52
*Turbo marmorata*	Turban shell	4.2 MU g^−1^ whole		Ishigaki Island, Japan	52

* MU = mouse units (1MU = 0.18 μgSTX)

** Presumed, genus and species name not given by author.

**Table 3:** Shellfish collected from New England, USA, federal waters in 2006. All testing was done by H^3^STX receptor binding assay. Highlighted results indicate individuals above the action level (80 μg STX eq./100g tissue). M = male, F = female; LOD = below detection limit.

Sampling Date	Common Name	Scientific Name	Number of Animals	Sampling Coordinates	STX eq. (μg/100g)
7-8-06	Ocean Quahog	*Arctica islandica*	8	41 00.183N	7.2
				70 44.543W	
7-8-06	Ocean Quahog	*Arctica islandica*	3	41 06.476N	11.6
				70 27.150W	
7-9-06	Whelk	*Busycon sp.*	3	41 25.057N	234.3
				70 02.751W	
7-9-06	Atlantic Surfclam	*Spisula solidissima*	3	41 25.057N	15.6
				70 02.751W	
7-9-06	Blue Mussels	*Mytilus edulus*	12	41 23.836N	19.5
				69 53.954W	
7-9-06	Blue Mussels	*Mytilus edulus*	12	41 23.836N	26.3
				69 53.954W	
7-9-06	Northern Moon Snail	*Lunatia heros*	3	41 26.084N	265.5
				70 03.000W	
7-9-06	Northern Moon Snail	*Lunatia heros*	7	41 23.836N	321.0
				69 53.954W	
7-10-06	Sea Scallops	*Placopecten magellanicus*	9	42 09.865N	228.8
				70 18.279W	
7-10-06	Sea Scallop viscera (F)	*Placopecten magellanicus*	1	42 09.865N	93.6
				70 18.279W	
7-10-06	Sea Scallop viscera (M)	*Placopecten magellanicus*	1	42 09.865N	131.9
				70 18.279W	
7-11-06	Ocean Quahog	*Arctica islandica*	11	42 12.025N	<LOD
				70 22.017W	
7-11-06	Sea Scallop	*Placopecten magellanicus*	6	42 11.391N	50.6
				70 19.700W	
7-11-06	Northern Moon Snails	*Lunatia heros*	6	42 11.391N	318.9
				70 19.700W	
7-11-06	Ocean Quahogs	*Arctica islandica*	12	42 12.025N	<LOD
				70 22.017W	
7-11-06	Blue Mussels	*Mytilus edulus*	9	42 12.025N	5.0
				70 22.017W	
7-11-06	Atlantic Surfclam	*Spisula solidissima*	2	42 11.391N	16.1
				70 19.700W	
7-11-06	Ocean Quahog	*Arctica islandica*	5	42 12.025N	12.0
				70 22.017W	
7-11-06	Ocean Quahog	*Arctica islandica*	4	42 11.391N	0.2
				70 19.700W	

* Number of whole animals homogenized to form representative sample.

** For sea scallops only combined viscera and gonad tested, unless otherwise indicated.

**Table 4:** Maximum STX concentrations, microalgal sources, and geographical reports of STXs in crustaceans.

Crustacean species and presumptive microalgal source	Common name	Maximum STX(s) concentration	Location	Reference
***Alexandrium catenella***				
*Cancer magister*	Dungeness crab	72 μg STX eq./100g viscera	Washington, USA	85
*Cancer productus*	Red rock crab	285 μg STX eq./100g viscera 27 μg STX eq./100g muscle	Washington, USA	161
*Fabia subquadrata*	Pea crab	32 μg STX eq./100g whole crabs	Washington, USA	85
*Hemigrapsus nudus*	Purple shore crab	44 μg STX eq./100g whole body minus legs and carapace	Washington, USA	161
*Hemigrapsus oregonensis*	Green shore crab	31 μg STX eq./100g whole	Washington, USA	161
*Pagurus* sp.	Hermit crab	35 μg STX eq./100g whole crabs	Washington, USA	85
*Pugettia producta*	Northern kelp crab	146 μg STX eq./100g eggs; 1710 μg STX eq./100g viscera; 48 μg STX eq./100g muscle	Washington, USA	161
*Balanus* spp.	Barnacles	84 μg STX eq./100g whole	Washington, USA	161

***Alexandrium tamarense***				
*Anonyx sarsi*	Gammarid amphipod	180 μg STX eq./100g (tissue not specified)	St.Lawrence estuary, Canada	162
*Cancer borealis*	Jonah crab	56 μg STX eq./100g (tissue not specified)	Maine, USA	85
*Homarus americanus*	American lobster	1512 μg STX eq./100g hepatopancreas (bioassay); 961 μg STX eq./100g hepatopancreas (HPLC); 69 μg STX eq./100g meat (HPLC)	Bay of Gaspe, Canada	162

***Pyrodinium bahamense***				
ND	Crab	339 MU* 100 g^−1^	Brunei Darussalam	141
ND	Mangrove crabs	239 MU 100 g^−1^ guts; 175 MU 100 g^−1^ gills	Sabah, Malaysia	138
*Portunus pelagicus*	Blue manna crab	175 MU 100 g^−1^ whole crab; 288 MU 100 g^−1^ gills; 328 MU 100 g^−1^ guts 1.8 μg STX eq./100g whole	Sabah, Malaysia Northwest Australia	138 147
*Panulirus versicolor*	Painted spiny lobster	175 MU 100 g^−1^ whole lobster; 175 MU 100 g^−1^ body only	Sabah, Malaysia	138
*Panulirus longipes*	Longlegged spiny lobster	211 MU 100 g^−1^ whole lobster; 177 MU 100 g^−1^ head and legs	Sabah, Malaysia	138
ND	Penaeid shrimp	175 MU 100 g^−1^ frozen tails; 268 MU 100 g^−1^ body only	Sabah, Malaysia	138
ND	Penaeid shrimp “Udang”	190 MU 100 g^−1^	Brunei Darussalam	141

**Unknown origin**				
*Hemigrapsus sanguineus*	Asian shore crab	0.16 MU g^−1^ hepatopancreas	Sanriku coast, Japan	146
*Metopograpsus frontalis*	Mangrove shore crab	10.0 μg STX eq./100g whole	Northwest Australia	147
*Pachygrapsus crassipes*	Striped shore crab	0.10 MU g^−1^ hepatopancreas	Sanriku coast, Japan	146
*Percnon planissimum*	Sally lightfoot crab	7.4 MU g^−1^ whole	Ishigaki Island, Japan	52
*Pilumnus pulcher*	Hairy crab	80 μg STX eq./100g whole	Northwest Australia	147
*Pilumnus vespertilio*	Hairy crab	120 μg STX eq./100g whole 6.1 MU g^−1^ whole	Northwest Australia Ishigaki Island, Japan	147 52
*Schizophrys aspera*	Eyelash spider crab	2.3 MU g^−1^ whole	Ishigaki Island, Japan	52
*Telmessus acutidens*	Edible shore crab	2723 μg STX eq./100g viscera	Fukushima Prefecture, Japan	163,164
*Actaeodes tomentosus*	Xanthid crab	130 MU g^−1^ whole	Ishigaki Island, Japan	52
*Atergatis floridus*	Xanthid crab	Positive STX, NEO, GTX2 16,611 μg STX eq./100g whole 490 MU g^−1^ whole Positive GTX 1–4	Fiji Islands Northwest Australia Ishigaki Island, Japan	165 147 52
*Atergatopsis germaini*	Xanthid crab	Positive GTX 3, NEO, STX	Taiwan	167
*Demania reynaudi*	Xanthid crab	Positive GTX 3–4, NEO	Taiwan	166
*Eriphia scabricula*	Xanthid crab	180 MU g^−1^ whole	Ishigaki Island, Japan	52
*Eriphia sebana*	Xanthid crab	Positive STX, NEO, GTX1, GTX2	Great Barrier Reef, Australia	168
*Euzanthus exsculptus*	Xanthid crab	29 μg STX eq./100g whole	Northwest Australia	147
*Lophozozymus octodentatus*	Xanthid crab	23 μg STX eq./100g whole	Northwest Australia	147
*Lophozozymus pictor*	Xanthid crab	18.9 MU g^−1^ whole crab Positive GTX	Australia Taiwan	169 170
*Neoxanthias impressus*	Xanthid crab	147 μg STX eq./100g whole 10 MU g^−1^ whole	Northwest Australia Ishigaki Island, Japan	147 52
*Platypodia granulosa*	Xanthid crab	110 MU g^−1^ whole	Ishigaki Island, Japan	52
*Platypodia pseudogranulosa*	Xanthid crab	10 μg STX eq./100g whole	Northwest Australia	147
*Xanthias lividus*	Xanthid crab	Positive GTX	Taiwan	171
*Zosimus aeneus*	Xanthid crab	Positive STX, NEOSTX, GTXI-3 Positive GTX 660 MU g^−1^ whole 108,000 μg STX eq./100g chelae muscle; 720 μg STX eq./100g cephalothorax muscle 78 μg STX eq./100g whole 259 MU g^−1^ whole crab	Fiji Islands Taiwan Ishigaki Island, Japan Japan Northwest Australia Philippines	165 171 52 172 147 173
*Procambarus clarkii*	Red swamp crayfish	0.23 MU g^−1^ hepatopancreas	Sanriku, Japan	146
*Carcinoscorpius rotundicauda*	Mangrove horseshoe crab	STX	Thailand	174

* MU = mouse units (1MU = 0.18 μgSTX); ND = no data

**Table 5:** Maximum STX concentrations, microalgal sources, and geographical reports of STXs in various fish tissues and species.

Fish species and presumptive microalgal source	Common name	Maximum STX(s) concentration	Location	Reference
***Alexandrium fundyense***				
* Scomber scombrus*	Atlantic mackerel	209 μg STX eq./100g liver; 367 μg STX eq./100g liver	Bay of Fundy; Gulf of St. Lawrence	194–195

***Alexandrium tamarense***				
* Scomber japonicus*	Chub mackerel	2800 μg STX eq./100g muscle; 500 μg STX eq./100g liver; 72 μg STX eq./100g gills	Argentina	196

***Pyrodinium bahamense***				
* Rastrelliger* sp.	Short mackerel	99 MU 100 g^−1^ tissue	Brunei Darussalam	141
* Sardinella* sp.	Sardinella	99 MU 100 g^−1^ tissue 572 μg STX eq./100g guts	Brunei Darussalam Sabah, Malaysia	141 139
* Sphoeroides nephelus*	Southern puffer fish	1,443 μg STX eq./100g liver; 14,571 μg STX eq./100g muscle	USA	58
* Sphoeroides testudineus*	Checkered puffer fish	51.1 μg STX eq./100g liver; 104.3 μg STX eq./100g muscle	USA	58
* Sphoeroides spengleri*	Bandtail puffer fish	364.5 μg STX eq./100g muscle	USA	58

**Unknown origin**				
* Cololabis saira*	Pacific saury	0.14 MU g^−1^ viscera	Iwate, Japan	146
* Gadus macrocephalus*	Pacific cod	0.10 MU g^−1^ viscera; 0.10 MU g^−1^ intestine	Iwate, Japan	146
* Lamna ditropis*	Salmon shark	0.17 MU g^−1^ liver	Iwate, Japan	146
* Oncorhynchus keta*	Chum salmon	1.53 MU g^−1^ liver; 0.69 MU g^−1^ viscera	Iwate, Japan	146
* Scarus* (*= Ypsiscarus*) *ovifrons*	Knobsnout parrotfish	0.26 MU g^−1^ liver; 1.58 MU g^−1^ intestine	Iwate, Japan	146
* Arothron firmamentum*	Starry toadfish	740 MU g^−1^ ovary	Japan	197
* A. hispidus*	White-spotted puffer	Positive STX in liver, muscle, skin, and intestine	Philippines	198
* A. mappa*	Map puffer	Positive STX in liver, muscle, skin, and intestine	Philippines	198
* A. manillensis*	Narrow-lined puffer	Positive STX in liver, muscle, skin, and intestine	Philippines	198
* A. nigropunctatus*	Black spotted puffer	Positive STX in liver, muscle, skin, and intestine	Philippines	198
* A. reticularis*	Reticulated puffer	Positive STX in liver, muscle, skin, and intestine	Philippines	198
* A. stellatus*	Starry toadfish	Positive STX in liver, muscle, skin, and intestine	Philippines	198
* Chelonodon patoca*	Milk-spotted puffer	22.0 MU g^−1^ muscle; 40 MU g^−1^ skin; 12.0 MU g^−1^ liver; 2.8 MU g^−1^ ovary (data shown as mean) Positive STX in liver, muscle, skin, and intestine	Bangladesh Philippines	199 198
* Colomesus asellus*	Amazon puffer	53.2 MU whole body	Brazil	200
* Takifugu pardalis*	Panther puffer	Positive for STX in liver	Japan	201
* T. poecilonotus*	Fine patterned puffer	Positive for STX in liver, ovary and digestive tract	Japan	202
* T. radiates*	Puffer	Positive for STX in liver	Japan	202
* T. vermicularis*	Purple puffer	Positive for STX in liver, ovary and digestive tract	Japan	202
* Tetraodon cutcutia*	Ocellated puffer	7.6 MU g^−1^ muscle; 20 MU g^−1^ skin; 6.0 MU g^−1^ liver; 5.6 MU g^−1^ ovary (data shown as mean) 182 MU 100 g^−1^ skin; 238 MU 100 g^−1^ muscle; 106 MU 100 g^−1^ liver	Thailand Bangladesh	199 203
* T. cochinchinensis* (as *T. fangi*)	Puffer	Positive for STX whole body	Thailand	204
* T. suvatii*	Arrowhead puffer	191 MU g^−1^ muscle; 230 MU g^−1^ skin; 174 MU g^−1^ liver; 117 MU g^−1^ egg	Thailand	205
* T. turgidus*	Brown puffer	<2 MU g^−1^ muscle; 37 MU g^−1^ skin; <2 MU g^−1^ liver; 27 MU g^−1^ ovary	Cambodia	206

* MU = mouse units (1MU = 0.18 μgSTX)

## Footnotes

1 Steidinger *et al.* (1980) [49] distinguished *P. bahamense* var. *compressa* from *P. bahamense* var. *bahamense* based on morphological, dimensional, and toxicological characteristics. *P. bahamense* var. *compressa* was reported to be the toxic variety responsible for Indo-Pacific PSP events while *P. bahamense* var. *bahamense* was reported to be non-toxic. Landsberg *et al.* (2006) [58] reported for the first time STX(s) production from *P. bahamense* in Florida, USA putatively responsible for several cases of SPFP. Badylak *et al.* (2004) [57] confirmed that the *P. bahamense* variety occurring in Florida waters still conformed to Steidinger *et al.* (1980) [49] description of *P. bahamense* var. *bahamense* except that it now appeared to be toxic. The description of *P. bahamense* var. *compressa* vs. *P. bahamense* var. *bahamense* is currently being re-evaluated (K. Steidinger, personal communication), therefore throughout the text *P. bahamense* without varietal designation is used.
